# Lengthy complex lumbar fusion surgery in high-risk elderly patient under spinal anesthesia: A case report

**DOI:** 10.1016/j.ijscr.2019.10.053

**Published:** 2019-10-30

**Authors:** Ryan A. Curto, Charles C. Edwards, Charles Lin, Charles H. Brown

**Affiliations:** a The Maryland Spine Center, Mercy Medical Center, 301 St. Paul Pl, Baltimore, MD 21202, United States; b Department of Anesthesiology, Mercy Medical Center, 301 St. Paul Pl, Baltimore, MD 21202, United States; c Department of Anesthesiology and Critical Care Medicine, Johns Hopkins University School of Medicine, Sheikh Zayed Tower, 1800 Orleans St., Baltimore, MD 21287, United States

**Keywords:** Spinal anesthesia, Geriatric, Lumbar fusion, Spine surgery, Case report

## Abstract

**Introduction:**

Spinal Anesthesia (SA) continues to be an emerging technique for lumbar fusion surgery in the elderly population. SA is an appealing option in the high-risk geriatric population for several reasons, including the potential for reduced systematic stress, reduced blood loss, and reduced post-operative delirium. The safe limits of spine surgery under SA remain undetermined.

**Presentation of case:**

The following case-study describes an elderly high-risk patient (ASA III) with severe spinal stenosis and degenerative scoliosis who presented with lower back and right leg pain and underwent a 3-level lumbar fusion surgery with spinal anesthesia. The procedure lasted 3 h and 44 min with sufficient anesthesia maintained throughout. The patient experienced minor post-operative complications, but had an excellent clinical outcome at 3-month follow-up.

**Discussion:**

Further research should be conducted to define the temporal limits of SA in elderly patients and the etiology of post-operative complications following lumbar fusion surgery under spinal anesthesia in the geriatric population.

**Conclusion:**

The case reported, herein, demonstrates the feasibility of SA in elderly patients undergoing lengthy complex lumbar surgeries who have been designated “high-risk” patients (ASA > II) and provides support for future investigation into surgical and anesthesia treatment options for geriatric high-risk patients presenting with complex lumbar spine pathologies.

## Highlights

• The longest reported lumbar surgery in an elderly patient under spinal anesthesia.
• Spinal Anesthesia is a feasible option for lengthy geriatric lumbar surgery.
• SA affords effective anesthesia with possibly lower physiological stress to some older patients.
• Spinal Anesthesia may have elongated efficacy in the elderly population.

## 1 Introduction

Spinal Anesthesia (SA) continues to be an emerging technique for lumbar fusion surgeries in the elderly population. Previous studies have examined only relatively simple lumbar surgeries under SA and fail to examine complex cases of “high-risk” geriatric patients [[1], [2], [3], [4], [5], [6], [7]]. It has been shown that SA administration versus general anesthesia (GA) in geriatric patients may provide several previously documented advantages such as reduced blood loss, reduced post-operative pain scores, shorter post-operative care stays, and lower rates of blood transfusion [8]. The limits of complexity and duration of lumbar spine surgery in geriatric patients, however, remain unknown. We present a case report of a “high-risk” (ASA III) geriatric patient with severe spinal stenosis and degenerative scoliosis who elected to undergo a multi-level lumbar fusion surgery under SA. The patient’s care was managed in a private practice and hospital setting. To the authors’ knowledge, this is the longest reported lumbar fusion surgery under SA in a geriatric “high-risk” patient.

This case has been reported in line with the SCARE criteria [9].

## 2 Presentation of case

A 73-year old female presented with in an outpatient private-practice setting with recurrent lower back and right leg pain, following an elective lumbar decompression surgery 6 months prior which had provided transient symptom relief. These symptoms persisted despite an epidural injection. The patient’s medical history included generalized osteoarthrosis, COPD, type II diabetes mellitus, seizure disorder, inflammatory disease of the breast, chronic coronary artery disease, cardiac surgery, and hypertension. At the time of surgery, the patient reported to be a current every day smoker. She had a pre-operative body mass index of 31.1 kg/m^2^. The patient was classified as ASA III at the time of surgery.

**Fig. 1:**
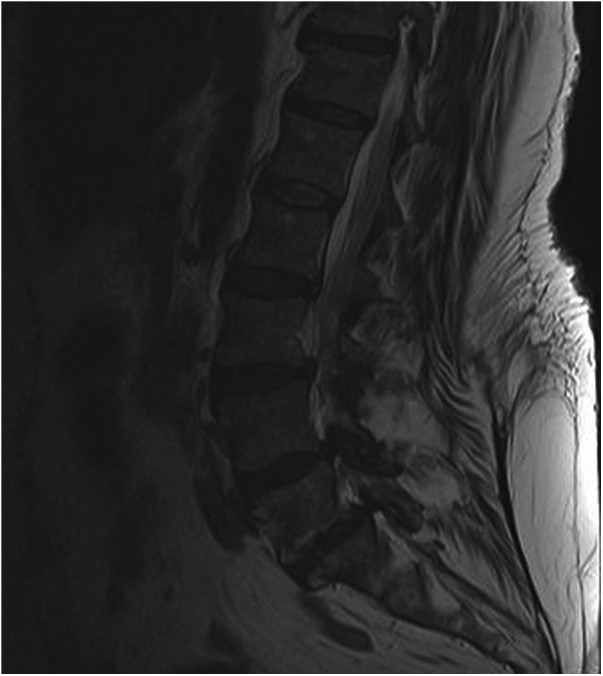
Pre-Operative Sagittal MRI.

A pre-operative MRI of the lumbar spine demonstrated severe recurrent lateral recess stenosis at L3-4, 4–5, and L5-S1 with synovial cysts, and adult degenerative scoliosis (Fig. 1). Due to the presence of gap facet signs suggesting apparent hypermobility and insufficiency of a previous decompression surgery to alleviate the patient’s symptoms, a revision decompression and L3-S1 lumbar fusion surgery with an interbody strut placement and iliac fixation was recommended. The patient elected to proceed accordingly. After review of pertinent comorbidities, the anesthesiologist and surgeon proceeded with the above-mentioned surgery utilizing spinal anesthesia.

Spinal Anesthesia was administered with the patient sitting in a forward hunched position. Using a 25-guage needle, 2.75 ml of 0.5 % Bupivacaine and 0.2 mg of morphine were injected to the intrathecal space at L3-4 to begin the spinal anesthesia. Intravenous fentanyl was administered at the beginning of the case (100 mcgs) for additional analgesia and then twice (50 mcg ×2) subsequently. Cefazolin (2 g) was administered intravenously to reduce the potential for infection, and Ephedrine (10 mg) was administered intravenously to prevent hypotension during the procedure. After administration, the patient was positioned prone on the Jackson table with pads (OSI, Union City, CA, USA). Sedation was achieved by Propofol infusion at a rate of 80 mcg/kg/min for the first 87 min. The patient was then administered Propofol at a rate of 100 mcg/kg/min for the remainder of the surgery.

The time from incision to closure was 3 h and 44 min. Estimated blood loss was 700 mL. The patient tolerated the procedure well without any known complications and was transported to the post-anesthesia care unit uneventfully.

**Fig. 2:**
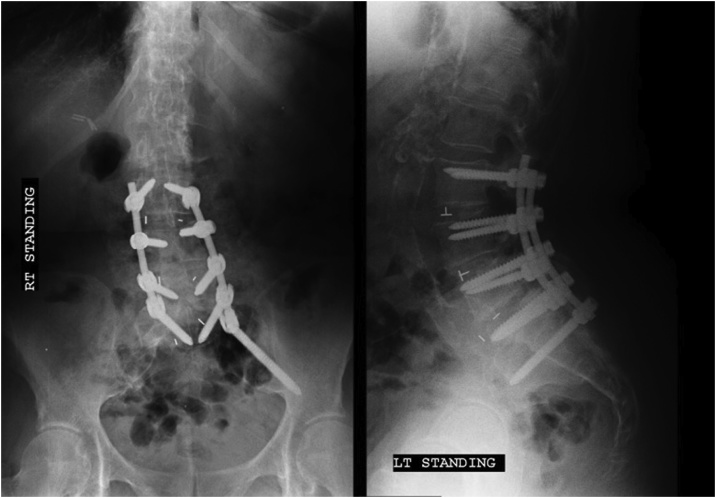
Post-Operative AP and Lateral X-rays.

The patient exhibited mild transient confusion on POD-2. This resolved following the temporary withholding of opioids. Postoperatively, patient had an HCT of 21.1 and was transfused 3 units of PRBCs. The patient’s HCT rose to 28.8 thereafter. After inpatient discharge on POD-4, the patient was transferred to a rehabilitation unit for 21 days before being discharged home. Following inpatient stay, patient continued without complications and excellent clinical progress at 3-month follow up (Fig. 2). The patient consented to the publication of her case.

## 3 Discussion

Many studies examining the characteristics of SA use in lumbar fusion surgeries focus on the middle-age population and less complex surgeries [[1], [2], [3], [4], [5], [6], [7]]. There remains a scarcity of published studies and reports focusing on complex lumbar spine surgeries in patients over 70 years old, especially those with high-risk comorbidities. One study focusing primarily on the geriatric population reported the case of an extended spine surgery (210 min) with use of SA [8]. In a separate report, Lessing et al. described a complex case of a 5-level posterior lumbar fusion using SA in a 72-year-old female lasting 214 min [10]. We present a 3-level lumbar fusion surgery in 73-year old high-risk patient (ASA III) performed under SA lasting 224 min.

As the half-life of Bupivacaine is 2.7 h, clinicians may question whether SA may be a viable option for procedures lasting longer than 3 h. Studies report that anesthetic medications may exhibit pronounced and extended effects in geriatric patients [11]. One such study reported a brachial plexus nerve root block provided sensory blockade for an average of 350 min in the elderly population compared to only 150 min in younger surgical population [12]. This may be attributed to altered pharmacokinetics and pharmacodynamics of an aging patient and may explain why satisfactory anesthesia was maintained throughout our patient’s procedure, herein reported.

Postoperative delirium is a concerning complication in elderly patients. Post-operative delirium has been shown to be a possible predictor of future cognitive and functional decline [13,14]· The patient in this case report experienced transient postoperative confusion. Although the confusion in this patient occurred after an increase in opioid administration and resolved after opioid discontinuance, the cause of confusion in this patient is not certain [15]. Several studies have suggested that reduced depth of anesthesia, as potentially provided by SA, could reduce the incidence of delirium although the literature is not consistent [[16], [17], [18], [19]]. Future high-level studies are needed to further examine the effect of anesthesia type and opioid administration on post-operative delirium, in particular, the role of SA.

Elderly patients may have decreased cardiovascular function in response to volumetric hematologic change [11]. It is therefore important to prioritize hemodynamic stability in the preoperative planning process of elderly high-risk patients. Finsterwald et al. provide evidence to suggest that SA in high-risk patients undergoing lumbar fusion surgery promotes hemodynamic stability, perhaps because of inhibited release of stress hormone intraoperatively [20]. Lessing et al. found that geriatric patients who underwent lumbar fusion surgery with SA had a lower rate of blood transfusion than their GA counterparts [8]. The elderly patient in this case required 3 units of blood during her inpatient stay due to a low HCT (21.1). Following the transfusion, the patient’s HCT returned to an acceptable level (28.8), and the patient was discharged without further complications. One could speculate whether the blood loss and need for transfusion would have been greater had general anesthesia been utilized.

We present a 73-year old patient female patient (ASA III) undergoing a successful three level lumbar fusion under spinal anesthesia for treatment of recurrent spinal stenosis, adult degenerative scoliosis, and lumbar radiculopathy. This surgery lasted 3 h and 44 min. To date this complex fusion procedure in a geriatric high-risk (ASA > II) patient is one of the longest spine surgeries ever performed under SA. While post-operative transient confusion and anemia occurred, it is unclear if these complications were related to the use of SA and the patient continued thereafter with excellent clinical progress.

## 4 Conclusion

This case report expands the published experience with SA in elderly high-risk patients undergoing spine surgery. Further investigation is required to clarify the upper limits of length and complexity for spine surgery in elderly patients undergoing SA.

## Sources of funding

This research did not receive any specific grant from funding agencies in the public, commercial, or not-for-profit sectors.

## Ethical approval

This case report is exempt from ethical approval by the Mercy Medical Center IRB.

## Consent

Written informed consent was obtained from the patient for publication of this case report and accompanying images. A copy of the written consent is available for review by the Editor-in-Chief of this journal on request.

Written and signed consent was obtained from the patient to publish this case report.

## Author’s contribution

Ryan A. Curto, B.A. – conception and design of study, acquisition of data, analysis and interpretation, drafting article, final approval of the version to be submitted.

Charles C. Edwards II, M.D. – conception and design of the study, acquisition of data, analysis and interpretation of data, critical revision of manuscript, final approval of the version to be submitted

Charles Lin, M.D. – acquisition of data, critical revision of manuscript, final approval of the version to be submitted.

Charles H. Brown IV, M.D. – conception and design of the study, critical revision of manuscript, final approval of the version to be submitted.

## Registration of research studies

N/A.

## Guarantor

Ryan A. Curto, B.A.

Charles C. Edwards II, M.D.

## Provenance and peer review

Not commissioned, externally peer-reviewed.

## Declaration of Competing Interest

Dr. Brown has consulted for and participated in a data share with Medtronic, unrelated to this manuscript. All other authors have no conflicts of interest to declare.
